# Swimming Exercise Changes Hemodynamic Responses Evoked by Blockade of Excitatory Amino Receptors in the Rostral Ventrolateral Medulla in Spontaneously Hypertensive Rats

**DOI:** 10.1155/2014/487129

**Published:** 2014-02-18

**Authors:** Cristiana A. Ogihara, Gerhardus H. M. Schoorlemmer, Maria de Fátima M. Lazari, Gisele Giannocco, Oswaldo U. Lopes, Eduardo Colombari, Monica A. Sato

**Affiliations:** ^1^Department of Physiology, Faculdade de Medicina do ABC (FMABC), Avenida Principe de Gales 821, Vila Principe de Gales, 09060-650 Santo Andre, SP, Brazil; ^2^Department of Physiology, Federal University of Sao Paulo (UNIFESP), Rua Botucatu 862, Vila Clementino, 04023-901 Sao Paulo, SP, Brazil; ^3^Department of Pharmacology, Federal University of Sao Paulo (UNIFESP), Rua Tres de Maio 100, Vila Clementino, 04044-020 Sao Paulo, SP, Brazil; ^4^Department of Pathology and Physiology, School of Dentistry, Sao Paulo State University (UNESP), Rua Humaita 1680, Centro, 14801-385 Araraquara, SP, Brazil

## Abstract

Exercise training reduces sympathetic activity in hypertensive humans and rats. We hypothesized that the swimming exercise would change the neurotransmission in the rostral ventrolateral medulla (RVLM), a key region involved in sympathetic outflow, and hemodynamic control in spontaneously hypertensive rats (SHR) and Wistar-Kyoto (WKY) rats. Bilateral injections of kynurenic acid (KYN) were carried out in the RVLM in sedentary- (S-) or exercised- (E-) SHR and WKY rats submitted to swimming for 6  weeks. Rats were *α*-chloralose anesthetized and artificially ventilated, with Doppler flow probes around the lower abdominal aorta and superior mesenteric artery. Injections into the RVLM were made before and after i.v. L-NAME (nitric oxide synthase, NOS, inhibitor). Injections of KYN into the RVLM elicited a major vasodilation in the hindlimb more than in the mesenteric artery in E-SHR compared to S-SHR, but similar decrease in arterial pressure was observed in both groups. Injections of KYN into the RVLM after i.v. L-NAME attenuated the hindlimb vasodilation evoked by KYN and increased the mesenteric vasodilation in E-SHR. Swimming exercise can enhance the hindlimb vasodilation mediated by peripheral NO release, reducing the activation of neurons with EAA receptors in the RVLM in SHR.

## 1. Introduction

The rostral ventrolateral medulla (RVLM) is a brain stem region which contains the presympathetic neurons that innervate the sympathetic preganglionic neurons in the spinal cord. The activity of RVLM neurons is critical for the regulation of resting sympathetic outflow and arterial pressure [1–3]. The activity of RVLM neurons is controlled by excitatory and inhibitory neurotransmitters and the imbalance of these mediators seems to contribute to pathophysiological states as hypertension [1, 2, 4–6]. The L-glutamate is considered an important neurotransmitter in the RVLM involved in the maintenance of high blood pressure in hypertensive states. The blockade of excitatory amino acid (EAA) receptors in the RVLM evokes a marked fall in arterial pressure in SHR and other rat models of hypertension but not in normotensive rats [7–9]. This suggested the existence of an increased tonic excitatory drive to RVLM neurons in hypertensive animals [8].

Moderate exercise reduces the diastolic and systolic arterial pressure in hypertensive humans and animals [10–12]. The heart rate seems to be reduced by low-intensity treadmill running in SHR, likely due to a decrease in the sympathetic tone to the heart [13]. This effect has been also attributed to causing the decrease in the cardiac output in low-intensity treadmill running in SHR and consequently to reducing the hypertension [14].

The swimming exercise model in rats has been used as an interesting tool to investigate cardiovascular, respiratory, and metabolic adaptations in the body, whose changes may vary according to the intensity of exercise, time of duration, and age at the onset of exercise [15–23]. The question whether the swimming exercise can be a factor of stress has been raised by different investigators; nevertheless, recent studies [24] have shown that restraint stressed rats submitted to 4 weeks of swimming have a reduced anxiety-like behavior. These findings suggested that the low-intensity swimming exercise for at least 4 weeks induces anxiolytic-like actions in stressed rats.

RVLM neurons projecting to the sympathetic preganglionic neurons in the spinal cord are tonically active and respond to different stimuli that affect arterial pressure [25]. The RVLM receives excitatory inputs and evidence suggests that commissural nucleus of the solitary tract (commNTS) neurons sends projections to the RVLM [26]. Inhibition of commNTS neurons decreases splanchnic sympathetic nerve activity and markedly reduces arterial pressure in SHR. Previous findings of our laboratory have shown that GABAergic inhibition of commNTS elicits an enhanced hindlimb vasodilation in SHR and Wistar-Kyoto (WKY) rats submitted to swimming exercise [27] compared to sedentary rats.

Although the peripheral mechanisms underlying the cardiovascular changes evoked by exercise training have been widely described [1, 4, 10, 13, 28–33], the central mechanisms involved in cardiovascular control that can be changed by swimming exercise are not well understood. As the RVLM receives inputs from commNTS neurons and the swimming exercise changes the regional vascular responses evoked by GABAergic inhibition of commNTS in exercised-SHR and WKY rats, we hypothesized that swimming exercise could change the neurotransmission in the RVLM. In order to test this hypothesis, this study investigated the hemodynamic responses evoked by blockade of EAA receptors in the RVLM in sedentary or exercised-SHR and WKY rats.

In addition, as previous studies [7–9] have shown that the blockade of EAA receptors in the RVLM in SHR reduces AP and the nitric oxide is very known for producing vasodilation [34], this study evaluated whether nitric oxide is involved or not in the regional vascular responses upon blockade of EAA receptors into the RVLM of rats maintained sedentary or submitted to exercise. We also investigated if the swimming exercise can change the nitric oxide synthase gene expression in the RVLM neurons in SHR and WKY rats.

## 2. Methods

### 2.1. Animals

Adult male SHR and age-matched WKY rats (250–300 g, 14–16 weeks old) were obtained from the central animal facility of the Federal University of Sao Paulo. Rats were housed in groups of 3 in plastic cages in an air-conditioned room (20–24°C) with a 12 : 12 h light-dark cycle, and had free access to standard chow pellets and water. All procedures performed were in accordance with the National Institutes of Health (NIH) Guide for the Care and Use of Laboratory Animals and were approved by the Animal Ethics Committee of the Federal University of Sao Paulo (protocol number 1566/06).

The tail cuff blood pressure and heart rate were measured before starting the exercise training and after 6 weeks of swimming exercise. All SHR used in this study were hypertensive at the beginning of exercise bouts. Tail arterial pressure and heart rate derived from ECG were carried out as previously described [27].

### 2.2. Exercise Training

Swimming pools were plastic cylinders with a diameter of 30 cm and 60 cm high and filled to a height of 50 cm with lukewarm water (30–34°C), one for each rat. Rats were subjected to daily swimming sessions for 6 weeks, 5 days/week, always between 10:00 and 12:00 a.m. The duration of the sessions gradually increased during the first days (first day: 10 min, second day: 15 min, third and fourth days: 30 min, and subsequent days: 1 h). After the first week, exercise intensity was increased by placement of a small weight (2% of the body weight) around the chest of the rat during swimming. This load was used previously by Sturek et al. [22] (1984). During the exercise period, the age-matched sedentary control group was placed in individual empty swimming pools and exposed to similar noise and handling.

### 2.3. Surgical Preparation

On the next day after the completion of the last exercise session, rats were anesthetized with 2% halothane in 100% O_2_, which was used during all the surgical procedures. Femoral artery and vein were cannulated for pulsatile arterial pressure (PAP) recording and infusion of drugs. Mean arterial pressure (MAP) and heart rate (HR) were derived from PAP signal. Alpha-chloralose (60 mg kg^−1^, i.v.) was used for maintenance of anesthesia while physiological variables were being recorded after halothane withdrawal. Animals were unresponsive to noxious toe pinch and maintained a steady level of arterial pressure. A supplementary dose of *α*-chloralose (20 mg kg^−1^, i.v.) was infused, according to the stability of MAP and HR. The trachea was cannulated and animals were artificially ventilated with 100% O_2_. Rectal temperature was maintained between 37 and 38°C.

Miniature Doppler flow probes (Iowa Doppler Products, Iowa City, IA) were placed around the lower abdominal aorta (1.3 mm of lumen) and the superior mesenteric artery (1.0 mm of lumen) through a midline laparotomy for indirect measurement of hindquarter and mesenteric blood flow, respectively. The probe wires were exteriorized through a small opening left in the sutured wound. The flow probes leads were connected to a Doppler flowmeter (Department of Bioengineering, The University of Iowa, Iowa City, IA) for indirect blood flow measurement. More details about the Doppler technique, including the reliability of this method for estimation of the blood velocity, have been previously described by Haywood et al. [35] (1981). Relative hindquarter and mesenteric vascular conductance were calculated as the ratio of Doppler shift and mean arterial pressure (MAP). Data were presented as percentage of change from the baseline [(final conductance − initial conductance/initial conductance) × 100]. The PAP, MAP, HR, and blood flow were digitalized and recorded in a MacLab (PowerLab 8SP System).

The animals were placed in a stereotaxic apparatus (David Kopf) with the incisor bar placed at −11 mm below the intra-aural line, and a partial craniotomy of the occipital bone was made. The dorsal surface of the brain stem was exposed. After recording of baseline arterial pressure and blood flow, 60 nL of 50 mM L-glutamate (L-glu) was injected into the RVLM bilaterally in order to find out the center of this area. The L-glutamate is an excitatory amino acid, which usually evokes pressor response when injected into the RVLM, and indicates that neurons involved in cardiovascular control are located in the spot of injection. After 20 min, 60 nL of 27 mM kynurenic acid (KYN) was injected in each RVLM. The KYN is an antagonist of excitatory amino acid receptors, which avoids the binding of L-glutamate on the receptors. The coordinates used for RVLM injections were 2.6 mm rostral to the *calamus scriptorius*, ±1.8 mm lateral from the midline, and 2.2 mm below the dorsal surface of the brain at the level of *calamus scriptorius*. Microinjections were made with glass pipettes (20 *μ*m tip diameter) coupled to a pressure injection apparatus (PicoSpritzer II). The injection volume was estimated by displacement of the fluid meniscus in the pipette, measured with a surgical microscope (DF Vasconcelos) with a calibrated reticule.

**Figure 1:**
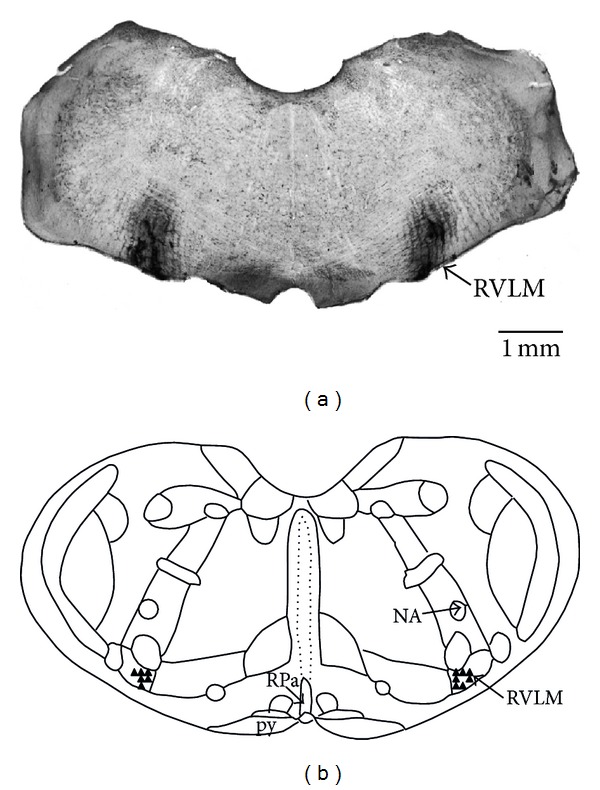
(a) Photomicrograph of a section of the medulla showing the site of bilateral injection of KYN into the RVLM marked with 4% Chicago Sky Blue dye (arrow) in one rat of the group. (b) Schematic representation of the sites of injections (triangles) in the RVLM at −12.48 mm from bregma [36]. NA: nucleus ambiguous, py: pyramidal tract, RPa: raphe pallidus, and RVLM: rostral ventrolateral medulla.

At the end of the experiments, the animals were deeply anesthetized with an overdose of i.v. sodium thiopental (170 mg kg^−1^) and 4% Chicago Sky Blue (Sigma Aldrich) dye was injected (60 nL) into the sites of drug injections. The animals were transcardially infused with 10% formalin solution. The brains were removed and maintained in 10% formalin for at least 24 h. The brain stem was cut in 40 *μ*m sections with a freezing microtome (Leica) and stained with 2% neutral red (Sigma Aldrich). The sections were analyzed with a light field microscope (Nikon) to verify the presence of Chicago Sky Blue dye in the RVLM.  Figure 1 shows a typical example of KYN injection sites in the RVLM (top) and depicts the site of dye deposition (−12.48 mm from bregma according to the atlas of Paxinos and Watson [36]) (2009) (bottom).

### 2.4. Data Analysis

Changes in the cardiovascular variables measured after L-glu or KYN injections into the RVLM were evaluated at the peak response. Results are expressed as mean±SE. Data were submitted to a two-way analysis of variance (ANOVA) followed by Tukey post hoc test for comparisons among the strains at the level of MAP, HR, percentage of change from baseline in hindquarter, and mesenteric conductances in anesthetized rats. Significance level was set at *P* < 0.05.

*Drugs.* Halothane (Tanohalo) and sodium thiopental were obtained from Cristalia Laboratory, Itapira, SP, Brazil; *α*-chloralose, kynurenic acid, and L-NAME were obtained from Sigma Aldrich (St. Louis, MO, USA). The vehicle used to dissolve *α*-chloralose was propylene glycol (Sigma, St. Louis, MO, USA). Kynurenic acid was prepared as previously described by Ito et al. [8] (2000). Sodium thiopental and L-NAME were dissolved in saline.

## 3. Results

### 3.1. Cardiovascular Changes Evoked by L-Glutamate and Blockade of Excitatory Amino Acid Receptors in the RVLM in Exercised and Sedentary SHR and WKY Rats

After anesthesia with *α*-chloralose, exercised (*N* = 6) and sedentary (*N* = 7) SHR showed similar resting MAP (162 ± 5 mmHg in exercised-SHR versus 172 ± 4 mmHg in sedentary SHR). Resting heart rate was significantly lower in exercised-SHR (295 ± 13 bpm) than in sedentary SHR (326 ± 10 bpm).

Bilateral injections of L-glu into the RVL elicited similar pressor responses in exercised and sedentary SHR or WKY rats. However, the pressor response evoked by L-glutamate was greater in exercised-SHR compared to exercised WKY rats (Table 1).

**Figure 2:**
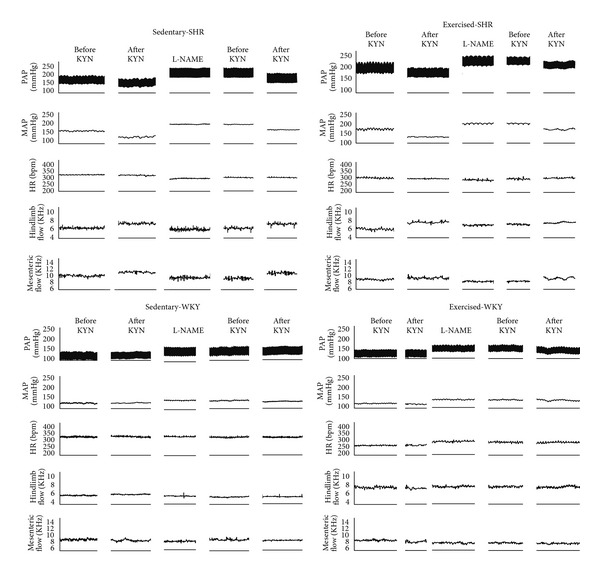
Tracings showing the effects of bilateral KYN microinjections into the RVLM before and after intravenous L-NAME infusion (25 *μ*mol/kg) on pulsatile arterial pressure (PAP, mmHg), mean arterial pressure (MAP, mmHg), heart rate (HR, bpm), hindlimb blood flow (KHz), and mesenteric blood flow (KHz) in sedentary- (S-) and exercised- (E-) SHR and WKY rats.

**Figure 3:**
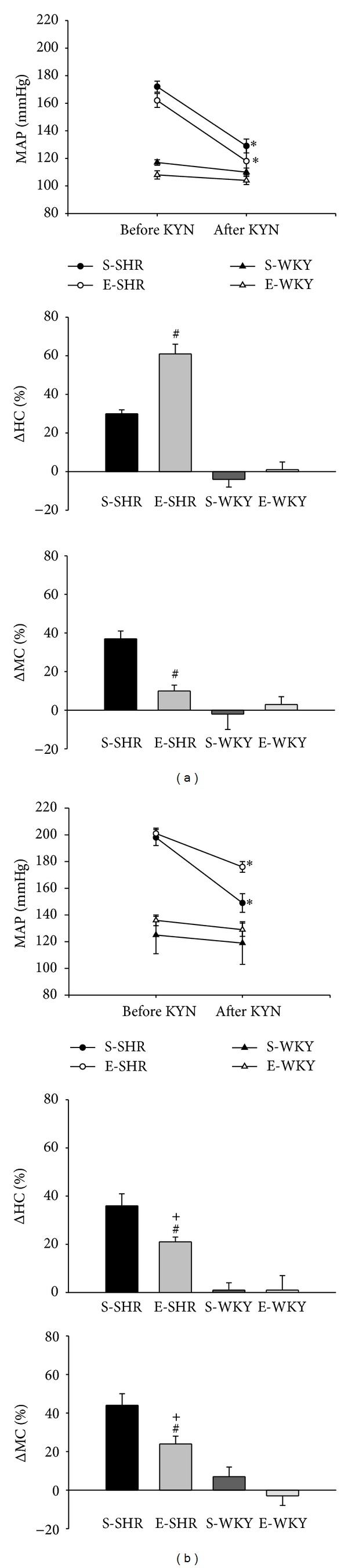
Mean arterial pressure responses (MAP, mmHg), percent change in hindquarter conductance (%ΔHC), and percent change in mesenteric conductance (%ΔMC) evoked by bilateral injections of KYN 27 mM (60 nL) into the RVLM in sedentary- (S-) and exercised- (E-) SHR and WKY rats before ((a) left panels) and after ((b) right panels) i.v. injection of L-NAME 25 *μ*mol/kg. *Different from before KYN, ^#^different from S-SHR, and ^+^different from E-SHR before i.v. L-NAME.

Bilateral injections of KYN into the RVLM elicited a marked decrease in MAP, both in exercised and sedentary SHR (−44 ± 7 mmHg in exercised versus −43 ± 5 mmHg in sedentary). Kynurenic acid in the RVLM did not change HR (−1 ± 21 bpm in exercised and −10 ± 11 bpm in sedentary). Nevertheless, KYN injections into the RVLM evoked a significant greater increase in the hindquarter conductance in exercised-SHR (61 ± 5%) compared to sedentary SHR (30 ± 2%). In contrast, exercised-SHR showed an attenuated increase in mesenteric conductance (10 ± 3%) compared to sedentary SHR (37 ± 4%) induced by KYN injections into the RVLM (Figures 2 and 3).

In WKY rats, after administration of *α*-chloralose, exercised (*N* = 6) and sedentary (*N* = 4) animals had similar resting MAP (108 ± 3 mmHg in exercised versus 111 ± 4 mmHg in sedentary-WKY rats) (Figure 2), but heart rate was lower in exercised (276 ± 9 bpm) compared to sedentary (368 ± 3 bpm) WKY rats. Injections of KYN into the RVLM of WKY rats neither induced hypotension (−4 ± 1 mmHg in exercised versus −8 ± 4 mmHg in sedentary) nor changed the hindquarter conductance (1 ± 4% in exercised versus −4 ± 4% in sedentary) (Figure 2). No difference was also observed in the mesenteric conductance after KYN injections into the RVLM in exercised (3 ± 4%) or sedentary (−2 ± 8%) WKY rats. Heart rate did not change after KYN into the RVLM in exercised (12 ± 10 bpm) and sedentary (10 ± 27 bpm) WKY rats (Figures 2 and 3).

### 3.2. Involvement of Nitric Oxide (NO) in the Cardiovascular Responses Evoked by Blockade of EAA Receptors in the RVLM in Exercised and Sedentary SHR and WKY Rats

In order to investigate if NO is involved in greater increase of hindquarter conductance observed in exercised-SHR after KYN injections into the RVLM or in greater increase of mesenteric conductance in sedentary SHR evoked by KYN injections into the RVLM, we compared the cardiovascular responses to KYN before and 30 min after intravenous injection of L-NAME, a nitric oxide synthase inhibitor (25 *μ*mol/kg). Similar changes in the HR were observed after i.v. L-NAME in sedentary and exercised-SHR or WKY rats (Table 2).

Although both exercised and sedentary SHR have showed greater MAP changes after i.v. L-NAME compared to WKY rats (Table 2), exercised-SHR showed an attenuated hypotension (−26 ± 2 mmHg) evoked by KYN injections into the RVLM compared to the response observed before i.v. L-NAME (−44 ± 7 mmHg) (Figure 3). No difference was observed in the HR responses elicited by KYN into the RVLM in sedentary and exercised-SHR after i.v. L-NAME in comparison to those observed before i.v. L-NAME.

Hindquarter conductance decreased more with i.v. L-NAME in sedentary SHR than in sedentary-WKY rats, while exercised-SHR and WKY rats showed similar decreases in hindquarter conductance after i.v. L-NAME (Table 2). Nevertheless, exercised-SHR had an attenuated increase in hindquarter conductance evoked by KYN injections into the RVLM after i.v. L-NAME (21 ± 2%) compared to the response before i.v. L-NAME (61 ± 5%). This increase in hindquarter conductance induced by KYN injections into the RVLM after i.v. L-NAME in exercised-SHR was also attenuated compared to sedentary SHR (Figure 3).

Exercised and sedentary SHR showed a greater decrease in mesenteric conductance after i.v. L-NAME compared to exercised and sedentary-WKY rats, respectively (Table 2). However, the increase in mesenteric conductance elicited by KYN injections into the RVLM was not different after i.v. L-NAME (44 ± 6%) compared to before i.v. L-NAME (37 ± 4%) in sedentary SHR. Exercised-SHR showed a greater increase in mesenteric conductance induced by KYN injections into the RVLM after L-NAME (10 ± 3%, Figure 3) in comparison to before L-NAME (24 ± 4%). In contrast, the increase in mesenteric conductance evoked by KYN injections into the RVLM after i.v. L-NAME was attenuated in exercised-SHR compared to sedentary SHR (Figure 3).

In WKY rats, KYN injections into the RVLM did not change hindquarter (1 ± 6% in exercised versus 1 ± 3% in sedentary) and mesenteric vascular conductances (−3 ± 5% in exercised versus 7 ± 5% in sedentary). The hemodynamic responses to KYN injections into the RVLM after i.v. L-NAME were similar to those observed prior to administration of i.v. L-NAME (Figure 3).

## 4. Discussion

The present study showed that after 6 weeks of swimming exercise, the blockade of EAA receptors in the RVLM evoked similar decrease in MAP in exercised and sedentary SHR and no significant changes in HR. These results extend the previous findings reported by Ito et al. [8] (2000) who suggested the existence of a tonic active excitatory input to RVLM in SHR but not in normotensive rats. Our findings also showed that the increase in hindquarter conductance evoked by blockade of EAA receptors with KYN injection into the RVLM in SHR is much increased by swimming exercise, suggesting a greater induced hindquarter vasodilation in these animals. In contrast, mesenteric conductance showed a smaller increase in exercised-SHR than in sedentary SHR after KYN injections into the RVLM, suggesting a reduced induced mesenteric vasodilation. Although the hemodynamic responses evoked by KYN into the RVLM were different comparing either exercised or sedentary SHR and WKY rats, the L-glutamate evoked similar pressor responses both in exercised and sedentary SHR. Additionally, despite the exercised and sedentary WKY rats showing similar responses to L-glutamate in the RVLM, the pressor response in exercised-SHR was enhanced in comparison to exercised WKY rats. These findings suggest that the swimming exercise can change the glutamatergic neurotransmission in the RVLM in SHR compared to WKY rats, but the protocol of swimming exercise used in this study was not able to reduce the high excitatory drive to the RVLM in SHR.

Previous study has reported that pressor responses in conscious rats evoked by L-glutamate in the RVLM are attenuated in swim-trained rats [23]. In contrast, L-glutamate in the RVLM produced similar pressor response in sedentary trained Sprague-Dawley rats anesthetized with Inactin, which were submitted to treadmill running, in spite of the attenuated lumbar sympathoexcitatory response to L-glutamate in trained rats [37]. The present study used a swimming exercise model for 6 weeks, which is different from exercise training protocols long lasting more than 8 weeks with higher intensity that induce metabolic changes. Therefore, in spite of the difference in anesthesia and rat strain among studies, it is likely that different adaptations in central cardiovascular control can be evoked by different approaches of exercise, intensity, and period of exercise training.

The blood flow measurement was not carried out in other vascular beds in addition to the hindlimb and mesenteric vessels in this study; indeed we cannot exclude the possibility that blockade of EAA receptors in the RVLM could induce changes in the conductance in other blood vessels in a differentiated manner comparing sedentary and exercised rats.

Although it is not fully understood if the RVLM has different sources of excitation, previous reports showed that the commNTS neurons arborize on RVLM and the GABAergic inhibition of commNTS neurons decreases splanchnic sympathetic nerve activity and MAP in SHR [26, 38]. In addition, GABA injection into the commNTS induces hindlimb vasodilation in exercised-SHR [27]. Inputs from muscle afferents release substance P into the NTS and activate GABAergic neurons [39, 40]. Evidence suggests that fibers from intermediate NTS pass through the commNTS or interneurons of the intermediate NTS projected to the commNTS, as lesions of the commNTS abolish the cardiovascular responses evoked by L-glutamate injected into the intermediate NTS [41]. It has been proposed that GABAergic neurons in the commNTS are mainly involved in depressor responses, a different role if compared with GABAergic neurons of the intermediate NTS, which seem to be more related to excitatory responses and also to the resetting of baroreceptor reflex mechanism toward high blood pressure during exercise [27, 40, 42–45]. The commNTS neurons are likely tonic active in SHR, but they can be inhibited by the signalization from intermediate NTS neurons receiving muscle afferents that seem to be necessary for the enhanced vasodilation in the hindlimb in rats submitted to swimming exercise [27]. Thereby, if the commNTS neurons provide excitation to the RVLM neurons, the exercise training is likely modulating the activity of commNTS and consequently the RVLM neurons. This could explain the enhanced hindquarter induced-vasodilation observed in exercised-SHR during blockade of EAA receptors in the RVLM. Conversely, this mechanism does not seem to be important in WKY rats, as the blockade of EAA receptors in the RVLM did not change the cardiovascular parameters. The absence of cardiovascular responses in WKY rats after blockade of EAA receptors in the RVLM is consistent with previous findings reported by Ito et al. [8] (2000).

Our study also showed that blockade of EAA receptors in the RVLM induced a smaller mesenteric vasodilation in exercised-SHR. Nevertheless, GABAergic inhibition of commNTS does not change the mesenteric conductance in both exercised and sedentary SHR or WKY rats [27]. Indeed, it is possible that commNTS neurons are not necessarily providing excitation to RVLM neurons to control the mesenteric blood flow. The smaller vasodilation observed by blockade of EAA receptors in the RVLM in exercised-SHR could be dependent on reduced excitation provided by other inputs to the RVLM, likely by other sources different from the commNTS. The selective regulation of regional blood flow by RVLM neurons has been previously proposed in other studies [46] and our findings suggest that swimming exercise can elicit a selective modulation of regional blood flow that can involve neurons with EAA receptors in the RVLM likely receiving inputs from different sources of excitation.

Previous study showed that blockade of EAA receptors in the RVLM elicited small increases in lumbar sympathetic nerve activity and arterial pressure either in sedentary or treadmill exercised Sprague Dawley rats [37]. In our study, blockade of EAA receptors did not produce any significant change neither in MAP nor in hindlimb and mesenteric conductances in normotensive WKY rats. A possible explanation for the different responses observed can lie in the fact that the anesthetics used in the current study were different from previous investigations [37] that used isoflurane and i.v. Inactin to anesthetize the animals. Another difference is the dose and volume of KYN injected into the RVLM. The previous study [37] used KYN at the dose of 40 mM and the volume injected into each RVLM was 90 nL, while in our study we used 27 mM of KYN and the volume of injection was 60 nL in each RVLM. Further, the strains of the rats used in both studies were different, and we cannot exclude the possibility that they can eventually show different cardiovascular responses even though both strains are considered normotensive rats.

As the blockade of EAA receptors in the RVLM in this study evoked an enhanced hindlimb vasodilation and a reduced mesenteric vasodilation in exercised-SHR, we investigated the role of nitric oxide in these hemodynamic responses. After intravenous infusion of L-NAME, the hindlimb vasodilatation evoked by blockade of EAA receptors in the RVLM was attenuated in exercised-SHR, but the mesenteric vasodilation was increased. This effect on the mesenteric bed could be a hemodynamic effect due to the shift of the blood flow toward this vessel during the reduced hindlimb vasodilation evoked by KYN injections into the RVLM after i.v. L-NAME. Even though the findings suggest that nitric oxide is involved in the hindlimb vasodilation evoked by KYN injections into the RVLM, we cannot also exclude the possibility that other vasodilatory factors different from nitric oxide could be released on the mesenteric vascular bed while the synthesis of nitric oxide was inhibited. In contrast, the absence of effect elicited by KYN injections into the RVLM on the hindlimb and mesenteric conductances in normotensive WKY rats remained unchanged after i.v. L-NAME.

The role of NO in control of the resistance of the hindlimb and mesenteric vascular beds has been demonstrated in previous studies. Electrical stimulation of the splanchnic nerve causes mesenteric vasoconstriction and simultaneous hindquarter vasodilatation. Hindquarter vasodilation was abolished after intravenous administration of L-NAME, suggesting that nitric oxide mediates this response [47]. Nitric oxide containing factors are also thought to mediate the hindlimb vasodilation evoked by electrical stimulation of the superior laryngeal nerve in rats [48]. Hypothalamic stimulation also causes a hindlimb vasodilation that is strongly reduced by i.v. administration of L-NAME [49]. Taken together, those studies strongly support a role of NO in the vasodilatory response in the hindlimb vascular bed.

In conclusion, our findings suggest that swimming exercise can change the hemodynamic responses dependent on blockade of excitatory amino acid receptors in the RVLM neurons in SHR. The inhibition of the RVLM neurons containing EAA receptors seems to be essential for the enhanced hindlimb vasodilatation in exercised-SHR, which is likely mediated by peripheral nitric oxide release.

To the best of our knowledge, no previous study has shown that swimming exercise for 6 weeks elicits changes in excitatory amino receptors in RVLM neurons inducing particularly a greater hindlimb vasodilation. Therefore, this study contributes to better understanding that even a low-intensity swimming exercise can selectively modulate the regional blood flow changing the activation of neurons with EAA receptors in the RVLM.

## Figures and Tables

**Table 1:** Mean arterial pressure (MAP, mmHg) and heart rate responses (HR, bpm) to microinjections of L-glutamate (60 nL, 50 mM) into the left and right RVLM in sedentary (S) and exercised (E) SHR and WKY rats (*N* = 6-7/group).

Group	MAP		HR	
	Left RVLM	Right RVLM	Left RVLM	Right RVLM
S-SHR	78 ± 13	66 ± 7	−61 ± 8	−52 ± 7
E-SHR	63 ± 4*	65 ± 3*	−70 ± 17	−71 ± 13
S-WKY	50 ± 5	59 ± 8	−53 ± 18	−49 ± 17
E-WKY	45 ± 5	46 ± 4	−68 ± 20	−69 ± 21

*Different from E-WKY.

**Table 2:** Mean arterial pressure (MAP, mmHg) and heart rate (HR, bpm) before and after i.v. L-NAME 25 *μ*mol/kg, change in mean arterial pressure (ΔMAP, mmHg), change in heart rate (ΔHR, bpm), and percent change in hindlimb (%ΔHC) and mesenteric conductances (%ΔMC) after i.v. L-NAME in sedentary (S) and exercised (E) SHR and WKY rats.

Variable	S-SHR	E-SHR	S-WKY	E-WKY
MAP before	161 ± 5	151 ± 3	108 ± 7	109 ± 4
MAP after	205 ± 4	203 ± 4	136 ± 6	141 ± 4
ΔMAP	43 ± 4*	52 ± 2^+^	28 ± 4	32 ± 3
HR before	331 ± 16	325 ± 11	389 ± 16	291 ± 28
HR after	293 ± 16	292 ± 9	335 ± 6	253 ± 19
ΔHR	−38 ± 5	−34 ± 6	−54 ± 12	−38 ± 13
%ΔHC	−53 ± 2%*	−48 ± 2%	−35 ± 4%	−48 ± 5%
%ΔMC	−52 ± 2%*	−50 ± 2%^+^	−40 ± 3%	−40 ± 3%

*Different from S-WKY.

^+^Different from E-WKY.

## References

[1] Hannum SM, Kasch FW (1981). Acute postexercise blood pressure response of hypertensive and normotensive men. *Scandinavian Journal of Sports Sciences*.

[2] Dampney RAL (1994). Functional organization of central pathways regulating the cardiovascular system. *Physiological Reviews*.

[3] Guyenet PG (2006). The sympathetic control of blood pressure. *Nature Reviews Neuroscience*.

[4] DiCarlo SE, Bishop VS (1988). Exercise training attenuates baroreflex regulation of nerve activity in rabbits. *The American Journal of Physiology*.

[5] Colombari E, Sato MA, Cravo SL, Bergamaschi CT, Campos RR, Lopes OU (2001). Role of the medulla oblongata in hypertension. *Hypertension*.

[6] Sved AF, Ito S, Sved JC (2003). Brainstem mechanisms of hypertension: role of the rostral ventrolateral medulla. *Current Hypertension Reports*.

[7] Bergamaschi C, Campos RR, Schor N, Lopes OU (1995). Role of the rostral ventrolateral medulla in maintenance of blood pressure in rats with Goldblatt hypertension. *Hypertension*.

[8] Ito S, Komatsu K, Tsukamoto K, Sved AF (2000). Excitatory amino acids in the rostral ventrolateral medulla support blood pressure in spontaneously hypertensive rats. *Hypertension*.

[9] Ito S, Komatsu K, Tsukamoto K, Sved AF (2001). Tonic excitatory input to the rostral ventrolateral medulla in Dahl salt-sensitive rats. *Hypertension*.

[10] Rupp H (1989). Differential effect of physical exercise routines on ventricular myosin and peripheral catecholamine stores in normotensive and spontaneously hypertensive rats. *Circulation Research*.

[11] de Sotomayor MA, Pérez-Guerrero C, Herrera MD, Marhuenda E (1999). Effects of chronic treatment with simvastatin on endothelial dysfunction in spontaneously hypertensive rats. *Journal of Hypertension*.

[12] Krieger EM, Justo da Silva GJ, Negrão CE (2001). Effects of exercise training on baroreflex control of the cardiovascular system. *Annals of the New York Academy of Sciences*.

[13] Gava NS, Veras-Silva AC, Negrao CE, Krieger EM (1995). Low-intensity exercise training attenuates cardiac *β*-adrenergic tone during exercise in spontaneously hypertensive rats. *Hypertension*.

[14] Véras-Silva AS, Mattos KC, Gava NS, Brum PC, Negrão CE, Krieger EM (1997). Low-intensity exercise training decreases cardiac output and hypertension in spontaneously hypertensive rats. *The American Journal of Physiology*.

[15] Barretti DL, Magalhaes FC, Fernandes T (2012). Effects of aerobic exercise training on cardiac renin-angiotensin system in an obese Zucker rat strain. *PLoS ONE*.

[16] Mastelari RB, Abreu SB, Corrêa FMA, de Souza HCD, Martins-Pinge MC (2012). Glutamatergic neurotransmission in the hypothalamus PVN on heart rate variability in exercise trained rats. *Autonomic Neuroscience*.

[17] Neto OB, Abate DTRS, Marocolo M (2013). Exercise training improves cardiovascular autonomic activity and attenuates renal damage in spontaneously hypertensive rats. *Journal of Science and Medicine in Sport*.

[18] Sakr HF (2013). Modulation of metabolic and cardiac dysfunctions by swimming in overweight rats on a high cholesterol and fructose diet: possible role of adiponectin. *Journal of Physiology and Pharmacology*.

[19] Radovits T, Olah A, Lux A (2013). Rat model of exercise-induced cardiac hypertrophy: hemodynamic characterization using left ventricular pressure-volume analysis. *The American Journal of Physiology*.

[20] Luiz RS, Silva KA, Rampaso RR (2013). Exercise attenuates renal dysfunction with preservation of myocardial function in chronic kidney disease. *PLoS ONE*.

[21] Endlich PW, Claudio ER, Gonçalves WLS, Gouvea SA, Moyses MR, de Abreu GR (2013). Swimming training prevents fat deposition and decreases angiotensin II-induced coronary vasoconstriction in ovariectomized rats. *Peptides*.

[22] Sturek ML, Bedford TG, Tipton CM, Newcomer L (1984). Acute cardiorespiratory responses of hypertensive rats to swimming and treadmill exercise. *Journal of Applied Physiology Respiratory Environmental and Exercise Physiology*.

[23] Martins-Pinge MC, Becker LK, Luccizano Garcia MR (2005). Attenuated pressor responses to amino acids in the rostral ventrolateral medulla after swimming training in conscious rats. *Autonomic Neuroscience*.

[24] Lapmanee S, Charoenphandhu N, Krishnamra N, Charoenphandhu J (2012). Anxiolytic-like actions of reboxetine, venlafaxine and endurance swimming in stressed male rats. *Behavioural Brain Research*.

[25] Guyenet PG, Loewy AD, Spyer KM (1990). Role of the ventral medulla oblongata in blood pressure regulation. *Central Regulation of Autonomic Functions*.

[26] Koshiya N, Guyenet PG (1996). NTS neurons with carotid chemoreceptor inputs arborize in the rostral ventrolateral medulla. *The American Journal of Physiology*.

[27] Ogihara CA, Schoorlemmer GHM, Levada AC (2010). Exercise changes regional vascular control by commissural NTS in spontaneously hypertensive rats. *The American Journal of Physiology*.

[28] DiCarlo SE, Bishop VS (1990). Exercise training enhances cardiac afferent inhibition of baroreflex function. *The American Journal of Physiology*.

[29] Arakawa K (1993). Hypertension and exercise. *Clinical and Experimental Hypertension*.

[30] Chandler MP, DiCarlo SE (1997). Sinoaortic denervation prevents postexercise reductions in arterial pressure and cardiac sympathetic tonus. *The American Journal of Physiology*.

[31] Krieger E-M, Brum P-C, Negrão C-E (1998). Role of arterial baroreceptor function on cardiovascular adjustments to acute and chronic dynamic exercise. *Biological Research*.

[32] Medeiros A, Oliveira EM, Gianolla R, Casarini DE, Negrão CE, Brum PC (2004). Swimming training increases cardiac vagal activity and induces cardiac hypertrophy in rats. *Brazilian Journal of Medical and Biological Research*.

[33] Tipton CM (2004). Exercise, training, and hypertension: an update. *Exercise and Sport Sciences Reviews*.

[34] Bhagat K, Vallance P (1996). Nitric oxide 9 years on. *Journal of the Royal Society of Medicine*.

[35] Haywood JR, Shaffer RA, Fastenow C, Fink GD, Brody MJ (1981). Regional blood flow measurement with pulsed Doppler flowmeter in conscious rat. *The American journal of physiology*.

[36] Paxinos G, Watson C (2009). *The Rat Brain in Stereotaxic Coordinates*.

[37] Mueller PJ (2007). Exercise training attenuates increases in lumbar sympathetic nerve activity produced by stimulation of the rostral ventrolateral medulla. *Journal of Applied Physiology*.

[38] Sato MA, Colombari E, Morrison SF (2002). Inhibition of neurons in commissural nucleus of solitary tract reduces sympathetic nerve activity in SHR. *The American Journal of Physiology*.

[39] Potts JT, Fuchs IE, Li J, Leshnower B, Mitchell JH (1999). Skeletal muscle afferent fibres release substance P in the nucleus tractus solitarii of anaesthetized cats. *Journal of Physiology*.

[40] Potts JT, Paton JFR, Mitchell JH (2003). Contraction-sensitive skeletal muscle afferents inhibit arterial baroreceptor signalling in the nucleus of the solitary tract: role of intrinsic GABA interneurons. *Neuroscience*.

[41] Colombari E, Menani JV, Talman WT (1996). Commissural NTS contributes to pressor responses to glutamate injected into the medial NTS of awake rats. *The American Journal of Physiology*.

[42] Catelli JM, Sved AF (1988). Enhanced pressor response to GABA in the nucleus tractus solitarii of the spontaneously hypertensive rat. *European Journal of Pharmacology*.

[43] Tsukamoto K, Sved AF (1993). Enhanced *γ*-aminobutyric acid-mediated responses in nucleus tractus solitarius of hypertensive rats. *Hypertension*.

[44] Mifflin SW (2001). What does the brain know about blood pressure?. *News in Physiological Sciences*.

[45] Zhang W, Herrera-Rosales M, Mifflin S (2007). Chronic hypertension enhances the postsynaptic effect of baclofen in the nucleus tractus solitarius. *Hypertension*.

[46] McAllen RM, Dampney RAL (1990). Vasomotor neurons in the rostral ventrolateral medulla are organized topographically with respect to type of vascular bed but not body region. *Neuroscience Letters*.

[47] Sato MA, Morrison SF, Lopes OU, Colombari E (2006). Differentiated hemodynamic changes controlled by splanchnic nerve. *Autonomic Neuroscience*.

[48] Possas OS, Lewis SJ (1997). No-containing factors mediate hindlimb vasodilation produced by superior laryngeal nerve stimulation. *The American Journal of Physiology*.

[49] Ferreira-Neto ML, Possas OS, Lopes OU, Cravo SL (2005). Evidence for a role of nitric oxide in hindlimb vasodilation induced by hypothalamic stimulation in anesthetized rats. *Anais da Academia Brasileira de Ciencias*.

